# Real-time data-driven motion correction in PET

**DOI:** 10.1186/s40658-018-0240-9

**Published:** 2019-01-09

**Authors:** Adam Kesner, C. Ross Schmidtlein, Claudia Kuntner

**Affiliations:** 1 Department of Medical Physics, Memorial Sloan Kettering Cancer Center, 1250 First Avenue, (Box 84), New York, NY 10065 USA; 2 AIT Austrian Institute of Technology, Seibersdorf, Austria

**Keywords:** Data-driven motion correction, PET, Real-time, Data-driven gating, Digital innovation

## Abstract

PET imaging has been, and continues to be, an evolving diagnostic technology. In recent years, the modernizing digital landscape has opened new opportunities for data-driven innovation. One such facet has been data-driven motion correction (DDMC) in PET. As both research and industry propel this technology forward, we can recognize prospects and opportunities for further development. The concept of clinical practicality is supported by DDMC approaches—it is what sets them apart from traditional hardware-driven motion correction strategies that have largely not gained acceptance in routine diagnostic PET; the ease of use of DDMC may help propel acceptance of motion correction solutions in clinical practice. As we reflect on the present field, we should consider that DDMC can be made even more practical, and likely more impactful, if further developed to fit within a real-time acquisition framework. This vision for development is not new, but has been made more feasible with contemporary electronics, and has begun to be revisited in contemporary literature. The opportunities for development lie on a new forefront of innovation where medical physics integrates with engineering, data science, and modern computing capacities. Real-time DDMC is a systems integration challenge, and achieving it will require cooperation between hardware and software developers, and likely academia and industry. While challenges for development do exist, it is likely that we will see real-time DDMC come to fruition in the coming years. This effort may establish groundwork for developing similar innovations in the emerging digital innovation age.

## Background

Motion correction in PET imaging has long been of interest in the nuclear imaging community. In the last decade, the development of data-driven motion correction (DDMC) has opened new opportunities in PET image motion correction. This new class of software-based strategies performs patient motion characterization via analysis of raw acquisition data, rather than using external hardware-based options. These motion characterizations can be integrated with entirely software-based motion correction workflows to generate motion-corrected images. Those working to further develop DDMC have pursued a shared vision that it may soon support a PET field where motion is inherently and routinely incorporated within standard imaging procedures [1]. The conceptual building blocks are well developed to support this future. Notably, performance characteristics, including accuracy, speed, and workflow integration, are readily improvable and continue to be improved upon. The limits of what can be achieved in these aspects have yet to be fully realized.

Early works demonstrating the potential of fully automated DDMC were published in 2007–2009 [2–4]. These efforts provided the foundational concept of this subfield: that significant motion information can be found in fluctuations of raw FDG-PET data. In 2010, our group published an article discussing potential of a real-time DDMC system and demonstrated this can be achieved by collapsing pre-reconstructed data space into more manageable data structures [5]. Since then, there have been numerous additions and contributions to developing DDMC ([6–11]). These articles demonstrate that robust DDMC is possible, though its practical, clinical implementation remains a systems integration challenge. Systems integration is traditionally in the purview of the vendor. Subsequently, the process of DDMC implementation, including real-time processing, has not been well studied in academic literature beyond discussion of potential.

## Main text

Motion correction in PET has shown promise for improving diagnostic and therapy applications [12–14]. Despite its promise, and more than a decade of research, it has not yet transitioned to routine application and standard-of-care diagnostic imaging. Image degradation from motion artifacts persists in the contemparary, state-of-the-art field.

A PET scanner is a highly sensitive instrument, made of tens of thousands of 511 KeV-sensitive detector elements that constantly monitor a large field of view with pico-second event timing resolution. These aspects make PET an ideal technology for DDMC. DDMC is a practical approach to the challenge of motion correction—practicality is its most distinguishing characteristic. The notion of real-time DDMC, as opposed to published post-processing DDMC methods, represents innovation in the speed of processing front. The innovation has appeal because it adds an additional layer of practicality to the technology. Currently, the only cost in inconvenience and/or efficiency of fully automated motion characterization is processing time; real-time methods offer promise to negate this, redefining patient motion characterization as an inherent aspect of our data, generated with insignificant background processing, and robustly available for clinical solution development. Beyond the practicality gained from removing motion tracking hardware from standard practice, discussed in previous publications [1, 5], we can envision other areas that may benefit from innovation in motion characterization processing time:

- Prospective gating in PET/CT: Real-time DDMC may support opportunities for improved complimentary modality image acquisitions in multimodality imaging via prospective motion gating. DDMC can be used in CT gating [15], and possibly for prospective CT gating that reduces patient dose [16]. Motion characterization of the PET can also be used for alignment of the PET and CT acquisition phases, to improve the attenuation correction and co-registration of anatomy.
- Prospective gating in PET/MR: Modern PET/MR system has geometry for simultaneous PET and MR acquisition. If motion is tracked in real-time via PET electronics, then this motion information can be integrated with MR signal acquisition and used to reduce motion artifacts in MR images. It may also eliminate the necessity to dedicate MR acquisition time to motion detection, thereby reducing overall acquisition time.
- Conformal PET acquisition protocols (PET acquisition time): Motion and motion correction are both largely patient-specific. Patients move to different extents with different motion patterns, and the ability to efficaciously correct their motion is also variable, on a scan-by-scan basis. A real-time motion characterization signal could be integrated with acquisition protocols such that patients who may benefit from motion correction receive longer scans and/or longer selective bed position dwell times to achieve greater image statistics and improved motion correction.
- Prospective conformal data sorting in PET: Motion correction is commonly employed via sorting data based on a patient’s motion characterization, and the sorting windows, for example, an “optimal bin,” are usually generally defined and/or based on population-derived motion characteristic studies. On the other hand, real-time motion characterization can enable full-time motion assessment, which can be used for data-driven conformal sorting [17]. This approach implements data-driven processing after acquisition of the motion signal and has the advantage of being a more patient/scan-specific solution.
- Radiotherapy: Motion correction is often used during treatment planning and treatment delivery. A real-time DDMC method/product could enable congruence of DDMC motion characterization across scanners, track internal, rather than surrogate motion during treatment planning, or possibly integrate PET-detected motion with therapeutic radiation delivery—like approaches used by RafleXion Medical (Hayward, CA, USA) [18].
- Assessment of patient motion: General assessment of patient motion for nuclear imaging could provide a useful measure of a patient state indicating the ability of the system to image reliably and reproducibly (e.g., inform on the detection of small lesions or max SUV for response assessment).
- Third party tools: Vendor neutral real-time DDMC tools could be developed, analogous to existing hardware motion tracking systems, and can support standardized motion characterization across users and systems. Non-vendor-specific products could allow for comparability of approaches and subsequently bolster competitive incentives for further innovation.

We have seen recent literature revisiting interest in fast, real-time processing for DDMC. Works by Salomon et al. [19] and Feng et al. [10] both present novel methods and discuss real-time processing motivation and implications. These works do not technically demonstrate real-time solutions but explore new fast methods that could potentially form the basis of a real-time application. Their work adds to a body of literature by groups who have achieved similar accomplishments [10, 20–23]. At the time of submission, the integration of real-time DDMC methods, by which we mean those methods that provide processed data at shorter times than can be seen in relevant physiological changes (e.g., motion, flow, uptake, clearance), have  yet to be realized. However, we do see indications of vendors developing this technology [24]

The reason it is taking so long to realize real-time DDMC tools may be that we are lacking the appropriate infrastructure to support its development. Academic and third party DDMC solution progress has been hindered by the issue of restricted data access [25]. The electronic infrastructure we have had in place over the last decades generally delineates pre-reconstructed data as proprietary [26]. This may be understandable given the considerable time, effort, and liability the vendors invest in developing these systems, but it does represent a fundamental bottleneck in developing and sharing new data-driven innovation. In this type of innovation, raw patient data is the resource being developed. Subsequently, access to data and data acquisition systems is required [27]. Data access, ownership, archiving, and usage liabilities are all aspects of digital innovation and need support from a modernized framework if data-driven innovation, like real-time DDMC, is to be realized and developed to its potential [28].

In the PET data use landscape, vendors are making noticeable efforts to support cooperation with academia and open their imaging data for researchers. Projects like PETlink TM (supported by Siemens) now provide open-access descriptions of their PET data [29]. We add that vendors make similar documentation available to users within research agreement frameworks and/or provide research processing tools, such as Siemens’ E7 tools, GE’s PET reconstruction toolboxes [30], and Philips’ Luminary Site Support Package. Our own group has recently received vendor permission to share our own DDMC products, which we are making freely available, and can be used for study, for other researchers to benchmark performance of their own DDMC methods or to support development of innovations at other points of the motion correction workflow [31]. We have also recently seen an independent research group develop a framework for intercepting PET acquisition data for complimentary, fast processing [32]. In this work, Markiewicz et al. demonstrated how information gained from powerful real-time processing can support numerous areas of innovation. We are seeing that an evolving infrastructure in image acquisition data management, particularly with respect to fast processing, is enabling new fronts of practical and useful innovations, likely including real-time DDMC.

## Conclusion

Real-time DDMC methods will likely come to fruition. If/when that happens, the availability and deployment of such practical DDMC will be transformative for the field of PET and have significant industrial, clinical, and academic implications [1]. Successful advancement of DDMC will require a pathway for data-driven solutions to integrate with PET systems via practical workflows. As the field looks forward, we should identify and support opportunities for academic researchers and PET vendors to cooperate in developing systems integration research and clinically accessible solutions. Access to data, and the speed in which it is processed, and human/system interaction paradigms are concepts relevant for real-time DDMC and represent new forefronts of digital innovation that require adequate infrastructural support. Working together, we should now attend to building that infrastructure, so that our field aligns with and flourishes within the emerging digital innovation age.
